# Circulating micronutrients levels and their association with the risk of endometriosis

**DOI:** 10.3389/fnut.2024.1466126

**Published:** 2024-10-16

**Authors:** Yanna Zhang, Meng Li, Feifei Zhang, Jiaoya Lin, Hong Yuan, Qing Nian

**Affiliations:** ^1^Department of Blood Transfusion, Sichuan Provincial People’s Hospital, University of Electronic Science and Technology of China, Chengdu, China; ^2^Blood Research Laboratory, Chengdu Blood Center, Chengdu, China

**Keywords:** endometriosis, micronutrients, Mendelian randomization, gynecological disorder, treatment strategies

## Abstract

**Background:**

Endometriosis, a prevalent gynecological disease, has an unclear pathogenesis. Micronutrients play a crucial role in disease development, which has led to an investigation of their association with endometriosis.

**Methods:**

In this study, we analyzed the relationship between 15 micronutrients and endometriosis using both univariate and multivariate Mendelian randomization (MR) to assess the correlation. The results were validated using high-performance liquid chromatography (HPLC).

**Results:**

The univariate MR analysis indicated that vitamin B6 (OR = 1.7060, 95% CI: 1.1796–2.4672, *p* = 0.0045) and calcium (OR = 1.4834, 95% CI: 1.0747–2.0475, *p* = 0.0165) are associated with an increased risk of endometriosis. Higher intakes of vitamin B6 and calcium are associated with a greater likelihood of developing endometriosis. The MR Egger regression’s intercept term demonstrated no evidence of pleiotropy (*p* > 0.05) or heterogeneity (p > 0.05) in the SNPs for calcium and vitamin B6. In multivariate MR analysis, vitamin B6 (OR = 2.397, 95% CI: 1.231–4.669, *p* = 0.01) was linked to an increased risk of endometriosis, independently of other exposure factors. No significant heterogeneity (*p* = 0.831) or pleiotropy (*p* = 0.369) was observed in the genetic variation of endometriosis, affirming the reliability of the multivariate MR analysis. HPLC confirmed a significant increase in serum levels of vitamin B6 and calcium, aligning with the MR analysis findings.

**Conclusion:**

Vitamin B6 and calcium may be associated with this disease, with vitamin B6 potentially acting as an independent risk factor. Further research is essential to elucidate the role of micronutrients in disease, offering novel insights for prevention and treatment strategies.

## Introduction

Endometriosis, a hormone-dependent gynecological disease, is increasingly prevalent (1–3). Characterized by ectopic endometrial tissue growth, it causes inflammation and severe pain (4, 5). This condition substantially affects patients’ quality of life and fertility, but its mechanisms are not fully understood (6). Current treatments, mainly medication and surgery, have limitations: medications may not resolve the root cause, and surgery could irreversibly affect fertility (7). Therefore, there is an urgent need for novel therapeutic approaches to this gynecological condition. Micronutrients, including essential minerals and vitamins, are crucial for health and regulate various physiological processes (8). Recent studies have highlighted the link between micronutrients and diseases, seeking to understand endometriosis pathogenesis from this perspective. Further investigation could yield new insights for endometriosis prevention and management, potentially leading to innovative treatments (9, 10).

Micronutrients, including copper, calcium, carotene, folate, iron, magnesium, potassium, vitamins A, B6, B12, C, D, E, and zinc, are crucial for health and may influence endometriosis progression (11–13). Endometriosis, a common gynecological condition, is influenced by factors like genetics, environment, and lifestyle (14). Recent studies suggest a link between micronutrient intake and endometriosis (15). For instance, copper, pivotal in various physiological processes such as blood production and immune function, can impact immunity (16). A deficiency in copper might compromise immunity, potentially elevating the risk of endometriosis (17). Calcium, crucial for bone health, also affects female reproductive system functionality and could be associated with endometriosis onset (18, 19). Vitamin B6 and B12 are deemed significant in preventing endometriosis, given their roles in energy metabolism and nervous system maintenance (20). Insufficient levels of these vitamins may lead to metabolic disturbances, heightening susceptibility to illnesses. Vitamins C and E, potent antioxidants, may reduce cell damage and inflammation, potentially assisting in regulating endometriosis symptoms (21, 22). Additionally, zinc, an essential mineral, affects cellular division, immune response, and anti-inflammatory processes. Inadequate zinc levels can impair the immune system and inflammatory reactions, thereby increasing disease risks. A balanced micronutrient intake is crucial for preventing endometriosis and other gynecological issues (23). By adopting a well-rounded diet and incorporating necessary vitamins and minerals sensibly, one can effectively lower disease risks and ensure optimal reproductive system health (24). However, despite studies suggesting correlations, uncertainties regarding calcium–magnesium-endometrium interactions remain due to confounding variables.

Mendelian Randomization (MR) analysis is a precise epidemiological method that minimizes susceptibility to environmental or social confounding factors and reverse causal relationships (25). By utilizing genetic variations strongly linked to exposure as instrumental variables, this approach establishes associations between exposure and outcome with greater reliability (26). Two-sample MR analysis, leveraging GWAS (Genome-Wide Association Study) aggregated data, enhances the assessment of causal relationships while boosting statistical power (27). This study will employ a combination of MR analysis, GWAS data, and Finngen data to investigate the causal link between 15 micronutrients and endometriosis through multivariate MR analysis (28). Meanwhile, the results of MR analysis were detected using high-performance liquid chromatography to further verify the causal relationship between micronutrients and endometriosis.

Conducting thorough research on micronutrients’ correlation with endometriosis may reveal the disease’s pathogenesis. This exploration could provide a scientific basis for developing future prevention and treatment strategies. Delving into this field not only enhances comprehension of disease fundamentals but also introduces innovative avenues to enhance patients’ quality of life and safeguard fertility.

## Methods

### Study design

**Figure 1:**
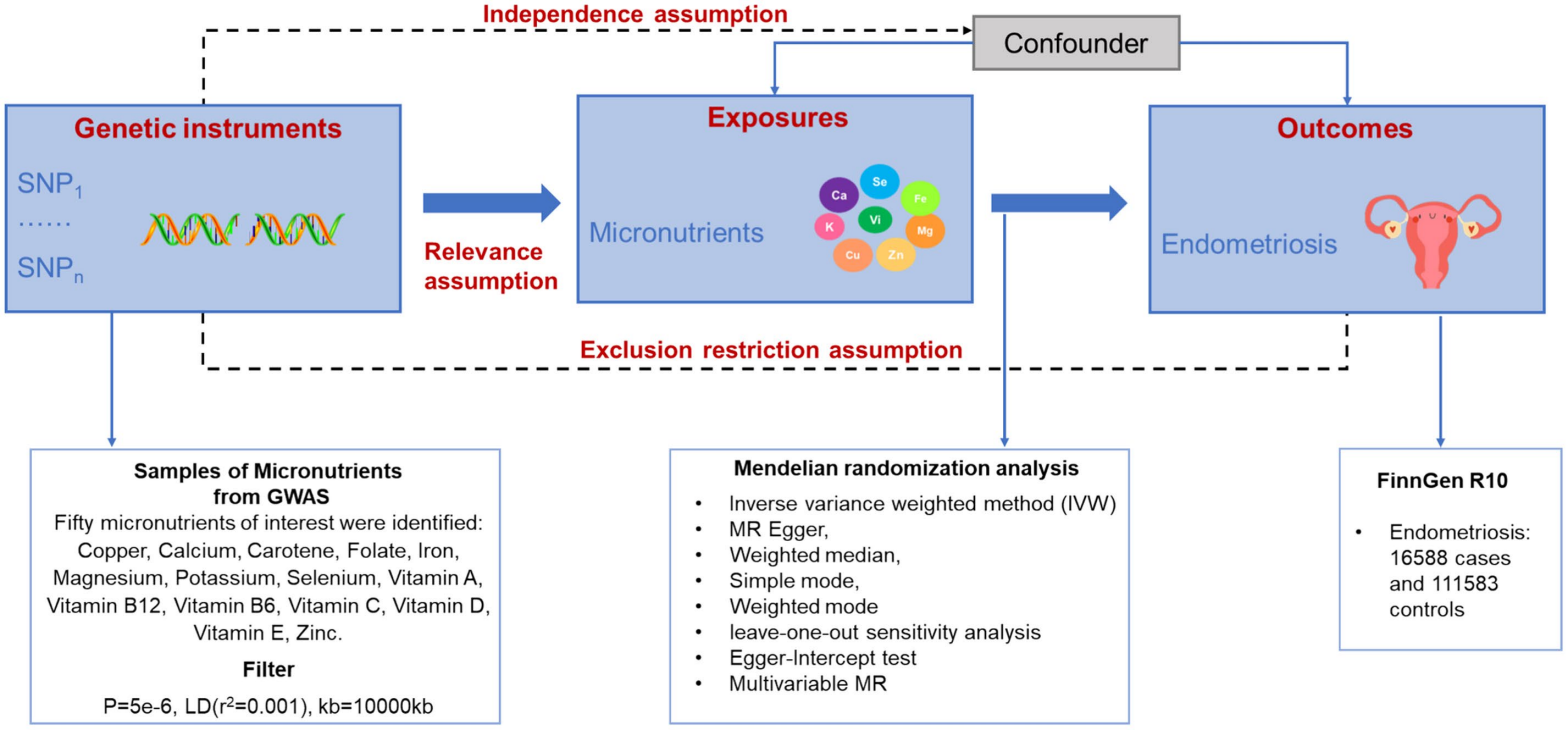
Overview of this bidirectional MR study design.

This study investigated the impact of 15 micronutrients on endometriosis, comprising 7 mineral micronutrients and 8 vitamins. The researchers employed the dual sample MR method to assess the causal relationships between these micronutrients and vitamins with endometriosis, as depicted in Figure 1. Stringent criteria were set to carefully select single nucleotide polymorphisms (SNPs) associated with the micronutrients and vitamins as instrumental variables to uphold the core hypothesis. During the selection of instrumental variables, SNPs linked to potential confounding factors were meticulously screened and excluded to ensure the assumption of independence was met. Moreover, to prevent instrumental variables from affecting outcomes through other pathways, a pleiotropy analysis was conducted during the MR analysis or instrumental variables showing signs of pleiotropy were removed to satisfy the exclusivity hypothesis (Supplementary Figure S1).

### Data selection and acquisition

In our study, we prioritized the GWAS catalog database^1^ and FinnGen database^2^ for their relevance to trace elements and endometriosis, and for their high data quality. Following a comprehensive literature review (29–32), we found these databases to be highly regarded for their extensive genetic and clinical data, including long-term follow-ups, and for their data quality, robustness, and credibility. Specifically, we sourced endometriosis genetic data from FinnGen database, which covers decades of longitudinal medical information and has a depth and breadth that is rare internationally. Concurrently, we collected trace element data from GWAS, a database rich in genetic markers and phenotypes, facilitating the study of genetic variations related to various traits and diseases. Both databases are regularly updated, and researchers can access and download the latest data for free through online platforms. During data selection, we applied stringent criteria based on epidemiological and genetic research standards, considering data reliability, sample size, representativeness, and accessibility to ensure the study’s transparency and reproducibility.

The summary data from genome-wide association studies for 15 micronutrients is sourced from the GWAS catalog database. These micronutrients include copper, calcium, carotene, folic acid, iron, magnesium, potassium, zinc, as well as vitamins A, B12, B6, C, D, and E. The information regarding endometriosis is obtained from the FinnGen database, as outlined in Supplementary Table S1.

### Instrumental variables selection

We identified significant single nucleotide polymorphisms (SNPs) (*p* < 5×10^−6^) from GWAS data for 15 micronutrients. The entire gene dataset from the European Thousand Genomes Project was utilized as a reference to assess linkage disequilibrium (LD) among SNPs. Independent SNPs (r^2^ < 0.001, distance >10,000 kb) were chosen as instrumental variables. To ensure that the selected SNPs were exclusively linked to exposure and outcomes, the PhenoScanner database was employed to scrutinize each SNP. To support the correlation hypothesis, we evaluated the strength of instrumental variables by calculating the F-statistic (*F* = *β*^2^/SE^2^), with β as the allele effect and SE as the standard error. An *F*-value greater than 10 indicates a robust association between instrumental variables and mineral micronutrients as well as vitamins. In cases where SNPs related to outcomes were missing, they were replaced with highly linked SNPs. SNPs without substitutes were excluded from the analysis.

### Univariate MR analysis

The IVW method was the primary research approach, with MR Egger, Weighted median, Simple mode, and Weighted mode analyses as supplements. IVW disregards intercept terms during regression and utilizes the reciprocal of outcome variance as weights for fitting (33, 34). Conversely, MR Egger regression considers intercept terms, displaying robustness and tolerance towards outliers and complex data factors, ensuring result accuracy and reliability. Weighted median estimation remains consistent even with up to 50% ineffective instrumental variables, addressing differences in instrumental variable accuracy effectively. Using median estimates for causal effects minimizes external interference, enhancing reliability. Simple mode adjusts for genetic variation impacts on exposure, eliminating confounders and reverse causal influences. Weighted mode enhances estimation efficiency and accuracy through genetic variation weighting, especially beneficial for large datasets, delivering precise causal effect estimates. Heterogeneity assessment via MR Egger and Inverse variance weighted analysis indicates homogeneity when *p* > 0.05. Multiplicity is assessed with the Egger Intercept test. Sensitivity analysis involves a leave-one-out method, sequentially removing each SNP to evaluate individual impact on merged results, ensuring overall result robustness. Funnel plots help detect bias presence. Applying these methods, we identified 15 micronutrients significantly associated with endometriosis.

### Multivariate MR analysis

By utilizing multivariate MR analysis (35), which combines MR principles with statistical methods such as multiple linear regression, we aim to determine if nutrients linked to endometriosis can each have distinct causal impacts. Additionally, we will conduct heterogeneity and pleiotropy analyses concurrently to assess the reliability and robustness of the findings.

### Determination of micronutrients content

With informed consent from endometriosis patients and healthy volunteers, we measured serum levels of vitamin B6 and calcium using high-performance liquid chromatography. Firstly, prepare a series of standard solutions containing micronutrients of different concentrations to establish a standard curve for quantitative analysis of the target substance. Then, inject the prepared serum sample into the HPLC instrument and analyze it under the specified conditions. Lastly, record and calculate the vitamin B6 and calcium levels.

### Statistical analysis

Statistical analyses were conducted using RStudio (version 4.3.0) and TwoSampleMR (version 0.5.7). Causal relationships between 15 micronutrients and endometriosis were established using Odds Ratios (OR) and 95% confidence intervals (CI). All statistical tests were two-tailed with a significance level set at *α* = 0.05.

## Results

### Filtering of instrumental variables

In this study, we acquired endometriosis genetic data from FinnGen databases, comprising 111,583 controls and 22,883 patients, along with 21,270,109 SNPs. Additionally, we sourced micronutrient genetic data from the GWAS database, including copper (*n* = 2,603, SNPs = 2,543,646), selenium (*n* = 2,603, SNPs = 2,543,617), zinc (*n* = 2,603, SNPs = 2,543,610), vitamin A (*n* = 460,351, SNPs = 9,851,867) and calcium, carotene, folate, iron, magnesium, potassium, vitamin B12, vitamin B6, vitamin C, vitamin D, and vitamin E, with a uniform sample size of 64,979 and 9,851,867 SNPs (Supplementary Table S1). We will select appropriate instrumental variables from these data based on the screening criteria of instrumental variables to explore the association between micronutrient and endometriosis. According to the instrumental variable screening criteria, the number of SNPs included in each nutrient is as follows: Copper: 6 SNPs, Selenium: 6 SNPs, Zinc: 8 SNPs, Folate: 12 SNPs, Carotene: 15 SNPs, Potassium: 14 SNPs, Iron: 11 SNPs, Calcium: 19 SNPs, Magnesium: 17 SNPs, Vitamin A: 11 SNPs, Vitamin B12: 8 SNPs, Vitamin B6: 17 SNPs, Vitamin C: 10 SNPs, Vitamin D: 13 SNPs, Vitamin E: 12 SNPs. Detailed SNP information is provided in Supplementary Table S2.

### Univariate MR analysis

**Figure 2:**
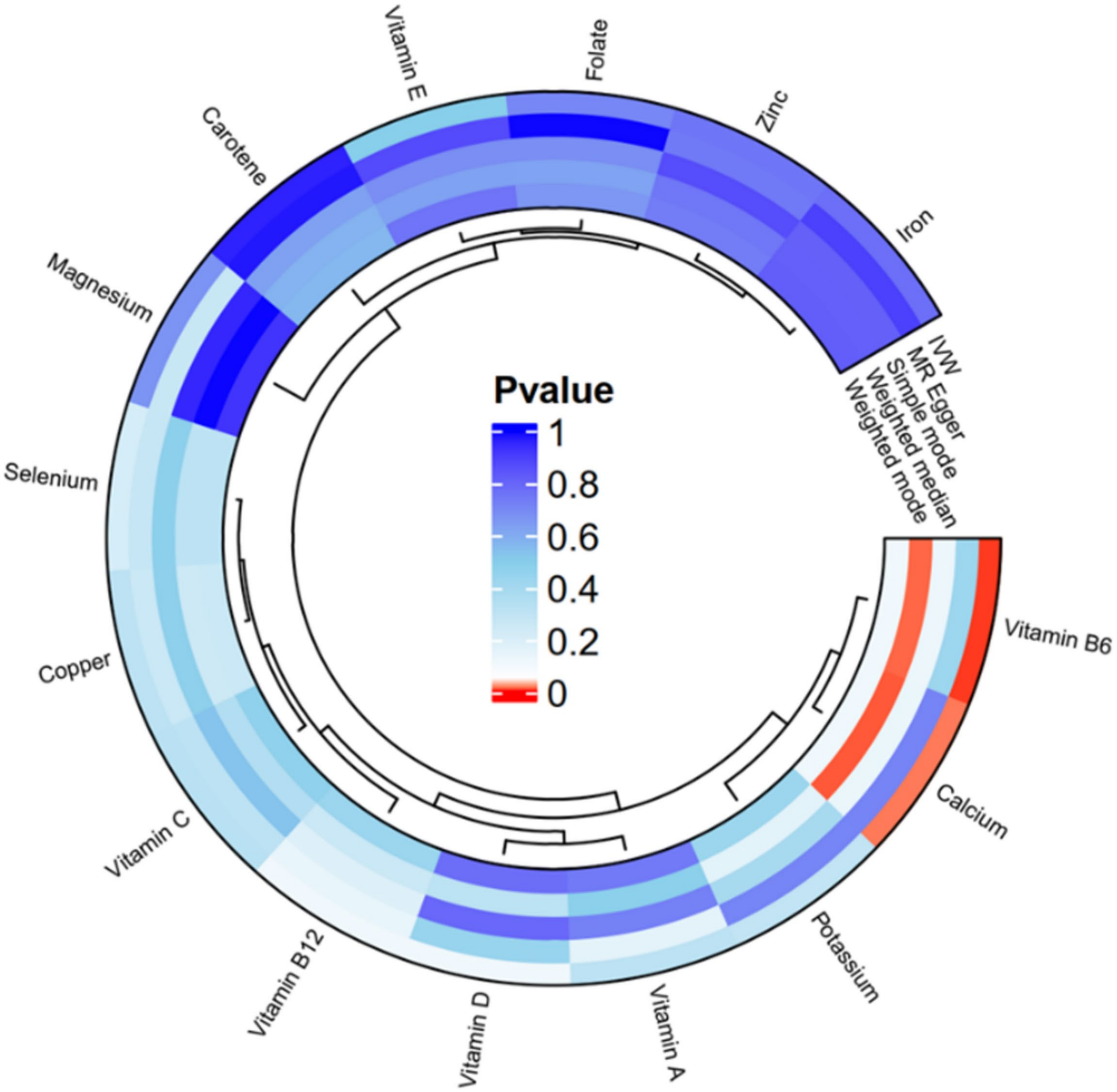
Circos plot of univariate MR analysis of 5 methods after removing heterogeneity with circulating micronutrients levels and endometriosis.

The IVW analysis in Figure 2 and Supplementary Table S3 reveal a significant causal link between calcium and endometriosis (OR = 1.4834, 95% CI: 1.0747–2.0475, *p* = 0.0165). This indicates that an excessive amount of calcium in women’s bodies heightens the risk of developing endometriosis. Similarly, there is a notable causal link between vitamin B6 and endometriosis (OR = 1.7060, 95% CI: 1.1796–2.4672, *p* = 0.0045), implying that elevated levels of vitamin B6 can increase the likelihood of endometriosis in women. On the other hand, Copper, Selenium, Zinc, Folate, Carotene, Potassium, Iron, Magnesium, Vitamin A, Vitamin B12, Vitamin C, Vitamin D, Vitamin E, and other micronutrients do not exhibit a causal relationship with endometriosis (*p* > 0.05). Additionally, the results from MR Egger, Weighted median, Simple mode, and Weighted mode methods align closely with those of the IVW method. Heterogeneity analysis performed using MR Egger and Inverse variance weighted (Supplementary Table S4) indicates no heterogeneity among the included SNPs (*p* > 0.05). The pleiotropy test of Egger Intercept test (Supplementary Table S4) reveals that horizontal pleiotropy does not introduce bias into the effect size of MR analysis (*p* > 0.05). Leave-one-out analysis demonstrates that no individual SNP significantly influences the effect size of MR analysis (Supplementary Figure S2). Collectively, these findings suggest the MR-derived causal relationship between micronutrients and endometriosis is reliable.

### MR analysis of endometriosis-related micronutrients

**Figure 3:**
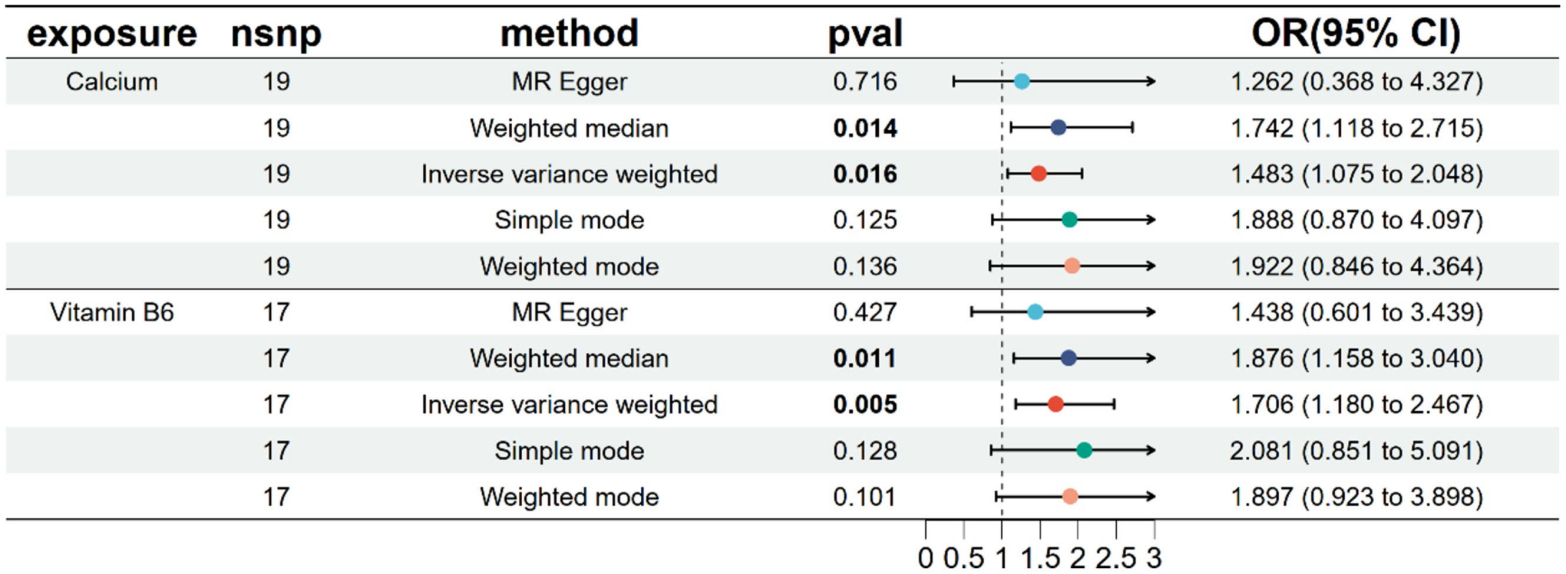
Univariate MR analysis of 5 methods after removing heterogeneity of endometriosis-related micronutrients.

A univariate MR analysis was conducted to investigate the causal effects of endometriosis-related micronutrients, specifically Calcium and vitamin B6, on endometriosis. The results revealed a significant causal relationship between calcium, vitamin B6, and endometriosis, as shown in Figure 3 and Supplementary Table S5. The intercept term of the MR Egger regression indicated no significant directed pleiotropy between SNPs in the Calcium and Vitamin B6 datasets, with *p*-values exceeding 0.05 (Table 1). No significant heterogeneity was detected in genetic variations related to calcium and endometriosis (Cochran’s Q = 13.084, *p* = 0.787) or vitamin B6 and endometriosis (Cochran’s Q = 19.838, *p* = 0.228) (Table 1). Consequently, inverse variance weighted (IVW) methods under fixed and random effects were separately employed to assess the causal relationship between Calcium, vitamin B6, and endometriosis. The IVW method indicated that higher Calcium levels was associated with an increased risk of endometriosis (OR = 1.4834, 95% CI: 1.0747–2.0475, *p* = 0.0165), and the same was true for vitamin B6 (OR = 1.7060, 95% CI: 1.1796–2.4672, *p* = 0.0045). The weighted median method, compared to the supplementary method, corroborated the impact of Calcium and vitamin B6 on the risk of endometriosis, underscoring the stability of the IVW method results (Figure 3). The scatter plot showcasing the potential impact of SNPs on Calcium, Vitamin B6, and endometriosis is depicted in Supplementary Figure S3, where the slope of each method signifies the magnitude of the effect evaluated by that specific method. Both individual and combined effects of Calcium and Vitamin B6 on endometriosis are illustrated in Supplementary Figure S4. Calcium exhibits 19 SNPs, and vitamin B6 has 17 SNPs associated with an increased risk of endometriosis (Supplementary Figure S5). The sensitivity analysis outcomes using the retention method are displayed in Supplementary Figure S6, indicating that no single SNP predominantly influences the overall effect.

**Table 1:** Heterogeneity and pleiotropy analysis for MR analysis of endometriosis-related micronutrients.

Exposure	Heterogeneity				Pleiotropy		
	Method	Q	Q_df	Q_pval	Egger intercept	se	pval
Vitamin B6	MR Egger	19.600	15	0.188	0.008	0.018	0.676
	Inverse variance weighted	19.838	16	0.228			
Calcium	MR Egger	13.013	17	0.735	0.006	0.021	0.793
	Inverse variance weighted	13.084	18	0.787			

### Multivariate MR analysis (MVMR)

**Figure 4:**
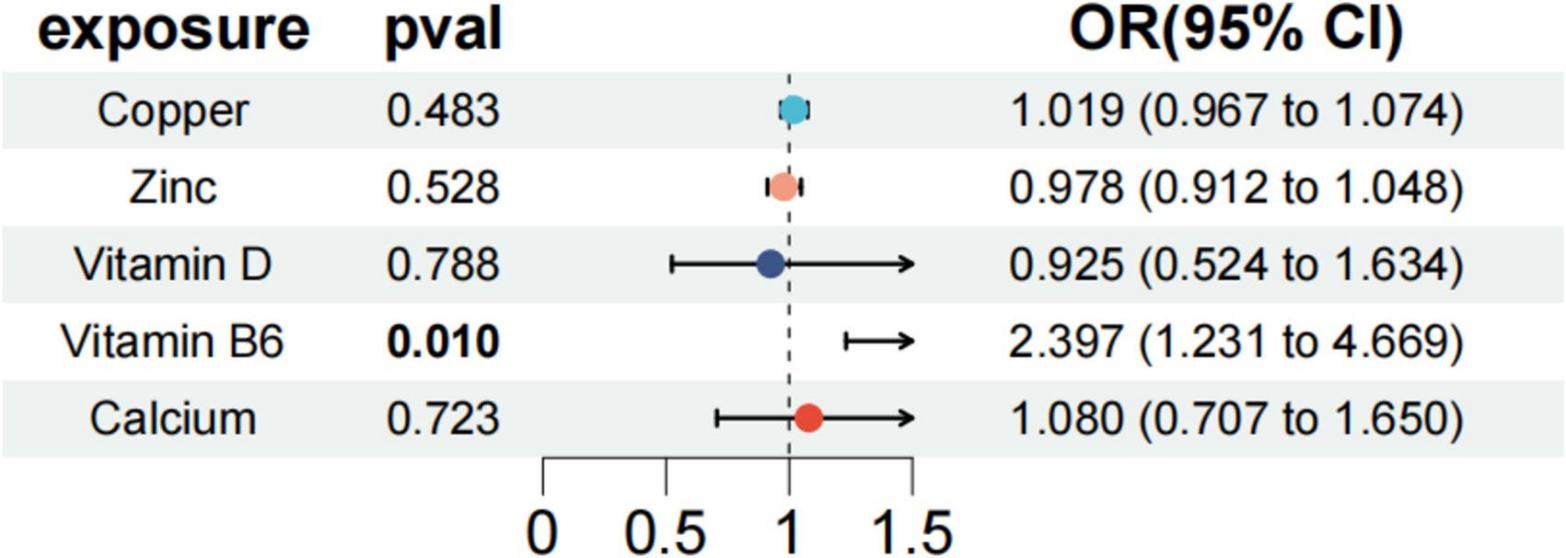
Multivariate MR analysis after removing heterogeneity of endometriosis-related micronutrients and endometriosis.

A multivariate MR analysis was conducted to explore the potential causal effects of micronutrients, including Vitamin D, copper, zinc, calcium, and vitamin B6, on endometriosis risk. The findings revealed that Vitamin B6 (OR = 2.397, 95% CI: 1.231–4.669, *p* = 0.01) independently correlated with other exposure factors, leading to an elevated risk of endometriosis (Figure 4). This indicates a significant role for vitamin B6 in endometriosis development. Moreover, the analysis found no significant heterogeneity (Cochran’s Q = 23.475, *p* = 0.831) or pleiotropy (*p* = 0.369) among the genetic variations associated with endometriosis (Table 2), supporting the credibility of the multivariate MR findings. Therefore, Vitamin B6 emerged as an independent factor associated with an increased risk of endometriosis, regardless of other exposure factors.

**Table 2:** Heterogeneity and pleiotropy analysis for multivariate MR analysis.

id.exposure	Exposure	β	se	pval	OR	Low 95%CI	High 95%CI	Heterogeneity		Pleiotropy	
								Q	*P*-value	Intercept	*P*-value
ieu-a-1073	Copper	0.019	0.027	0.483	1.019	0.967	1.074	23.475	0.831	−0.010	0.369
ieu-a-1079	Zinc	−0.022	0.036	0.528	0.978	0.912	1.048				
ukb-b-18593	Vitamin D	−0.078	0.290	0.788	0.925	0.524	1.634				
ukb-b-7864	Vitamin B6	0.874	0.340	0.010	2.397	1.231	4.669				
ukb-b-8951	Calcium	0.077	0.216	0.723	1.080	0.707	1.650				

### Evaluation of the assumptions of MR

To test the correlation hypothesis, we initially selected SNPs associated with 15 micronutrients from GWAS data with a substantial sample size. We applied a stringent genome-wide significance threshold of *p* < 5 × 10^−6^ and verified that the F-statistics for each SNP were above 10 (Supplementary Table S2). This rigorous selection process ensures the chosen SNPs are closely related to exposure, reducing the risk of weak instrument bias. Furthermore, to examine the assumption of independence, we utilized the PhenoScanner V2 tool to assess whether the SNPs were associated with any confounding factors or risk elements related to the 15 micronutrients. Our analysis indicated that none of the selected SNPs were linked to these confounding factors. Next, we performed MVMR, confirming consistent and reliable results (Figure 4 and Table 2). Lastly, to address the exclusion hypothesis, we grouped SNPs based on specific criteria to minimize LD and enhance their independence. Neither the MR Egger regression intercept nor the IVW method detected horizontal pleiotropy (*p* > 0.05), highlighting the robustness of these methods against such issues (Tables 1, 2 and Supplementary Table S4). Thus, estimates of causality are unlikely to be biased by this minor level of pleiotropy.

### The level of vitamin B6 and calcium in endometriosis and healthy volunteers

**Figure 5:**
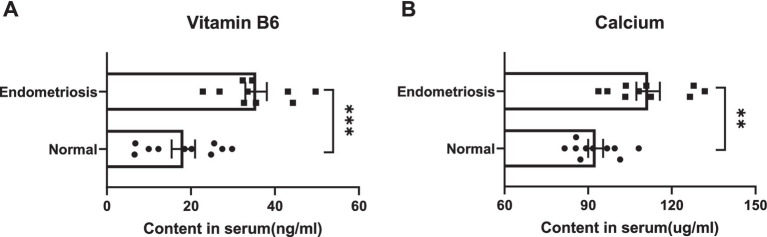
The content of micronutrients **(A)** Vitamin B6 and **(B)** Calcium in the serum of patients with endometriosis and healthy volunteers.

We detected the levels of Vitamin B6 and Calcium in the serum of patients with endometriosis and healthy volunteers using high-performance liquid chromatography. Compared with healthy volunteers, we observed a significant decrease in the levels of Vitamin B6 and Calcium in the serum of patients with endometriosis (Figure 5). These results indicate a significant correlation between the levels of Vitamin B6 and Calcium in serum and the occurrence of endometriosis. Additionally, our MR analysis further confirms this finding, confirming a significant association between serum levels of Vitamin B6 and Calcium and the occurrence of endometriosis. This research result provides new strategies and directions for the prevention, early diagnosis, and clinical treatment of endometriosis.

## Discussion

Endometriosis, a prevalent gynecological disease, poses a complex and not entirely elucidated pathogenesis (36). Micronutrients are pivotal in regulating disease onset, prompting our study to delve into the correlation between micronutrients and endometriosis (36, 37). By employing MR analysis and multivariate MR analysis, we aim to objectively assess the potential correlation between micronutrients and endometriosis.

In our study, we conducted a comprehensive analysis of the association between 15 micronutrients and endometriosis, utilizing GWAS data from FinnGen. Our analysis included the following micronutrients: copper, calcium, carotene, folate, iron, magnesium, potassium, vitamin A, B12, B6, C, D, E, and zinc. Using univariate MR analysis, we discovered a positive correlation between vitamin B6 and calcium intake and the risk of endometriosis. Specifically, higher intakes of vitamin B6 and calcium were associated with an increased likelihood of endometriosis. Our finding indicates that the intake of vitamin B6 and calcium could be linked to the onset of endometriosis. Concurrently, we employed high-performance liquid chromatography to measure serum vitamin B6 and calcium levels in both endometriosis patients and healthy volunteers. The results revealed significantly elevated serum levels of vitamin B6 and calcium in endometriosis patients, aligning with the MR analysis findings. Further evidence suggests that the association between vitamin B6 and calcium levels and the risk of endometriosis (20).

In addition, some research has highlighted vitamin B6 as an essential nutrient contributing to various metabolic pathways within the body, including amino acid metabolism and neurotransmitter synthesis (38–40). Further exploration is needed to ascertain whether its mechanism directly influences the pathogenesis of endometriosis. Conversely, calcium plays an essential role in physiological processes such as cellular signaling, muscle contraction, and neurotransmission (41–43). Excessive intake may disrupt calcium ion homeostasis, potentially contributing to health conditions like endometriosis (44, 45). These findings are consistent with our results, thereby reinforcing the hypothesis that links vitamin B6, calcium, and endometriosis.

Previous research has demonstrated a significant association between micronutrients, including vitamin D (46), copper (17, 47), and zinc (48), and the risk of endometriosis (20, 45). Our study further explores the relationship between these micronutrients—vitamin D, copper, and zinc—and endometriosis, incorporating a multifactorial MR analysis. Additionally, by identifying vitamin B6 and calcium as influential factors in univariate MR analysis, this this investigation reveals that vitamin B6 significantly influences endometriosis risk, independent of other factors. This novel finding highlights the unique role of vitamin B6 in endometriosis development, confirming previous research and strengthening the link between vitamin B6 and endometriosis.

However, it is worth noting that there are some limitations in this study. Firstly, we only relied on GWAS data and Finngen data for analysis, which may have resulted in incomplete consideration of other important factors. Future research should integrate additional data sources and comprehensive factors for more precise conclusions. Secondly, the intake of vitamin B6 and calcium may be influenced by other unmeasured potential factors, such as individual dietary habits and lifestyle, which may interfere with the results.

Despite its limitations, our study provides significant clinically relevant findings. First, we have uncovered new insights into the pathological mechanism of endometriosis. Vitamin B6 and calcium, identified as micronutrients linked to an elevated risk of this disease, may serve as potential predictive factors or targets for therapeutic interventions. Future investigations should thoroughly explore the roles of these micronutrients in endometriosis to craft more precise and efficacious prevention and treatment strategies. Second, our research outcomes lay a foundational groundwork for implementing pertinent public health initiatives. For instance, by propagating knowledge about the impact of vitamin B6 and calcium on endometriosis through educational campaigns, public awareness can be heightened. This, in turn, could guide individuals to adjust their daily dietary intake of these micronutrients accordingly. Furthermore, our study underscores the significance of micronutrients in the development of gynecological diseases, offering fresh perspectives for probing further into the connections between micronutrients and other such conditions. Given the vital importance of micronutrients in health, it is advisable to intensify relevant research efforts aimed at unraveling the mechanisms through which micronutrients influence gynecological diseases. This approach can significantly improve prevention and treatment of women’s health and gynecological disorders.

In summary, our findings indicate a potential association between vitamin B6, calcium and the risk of endometriosis. Furthermore, our study emphasizes vitamin B6’s crucial independent role in endometriosis development, providing new insights for prevention and treatment strategies. However, further research is essential to deepen our understanding of micronutrient mechanisms in endometriosis and to enhance the management of this common gynecological disorder.

## Data Availability

Publicly available datasets were analyzed in this study. This data can be found here: The data that support the findings of this study are openly available in IEU GWAS database at https://gwas.mrcieu.ac.uk/.

## Footnotes

^1^https://gwas.mrcieu.ac.uk/

^2^https://www.finngen.fi/en/access_results
