# Supplementary material for: Circulating micronutrients levels and their association with the risk of endometriosis
Source: Front Nutr. 2024 Oct 16;11:1466126. doi: 10.3389/fnut.2024.1466126 (PMC11521953; doi:10.3389/fnut.2024.1466126)
Supplement: Supplementary file 1 [file Data_Sheet_1.docx]

**Supplementary Figures**

**Figure S1**


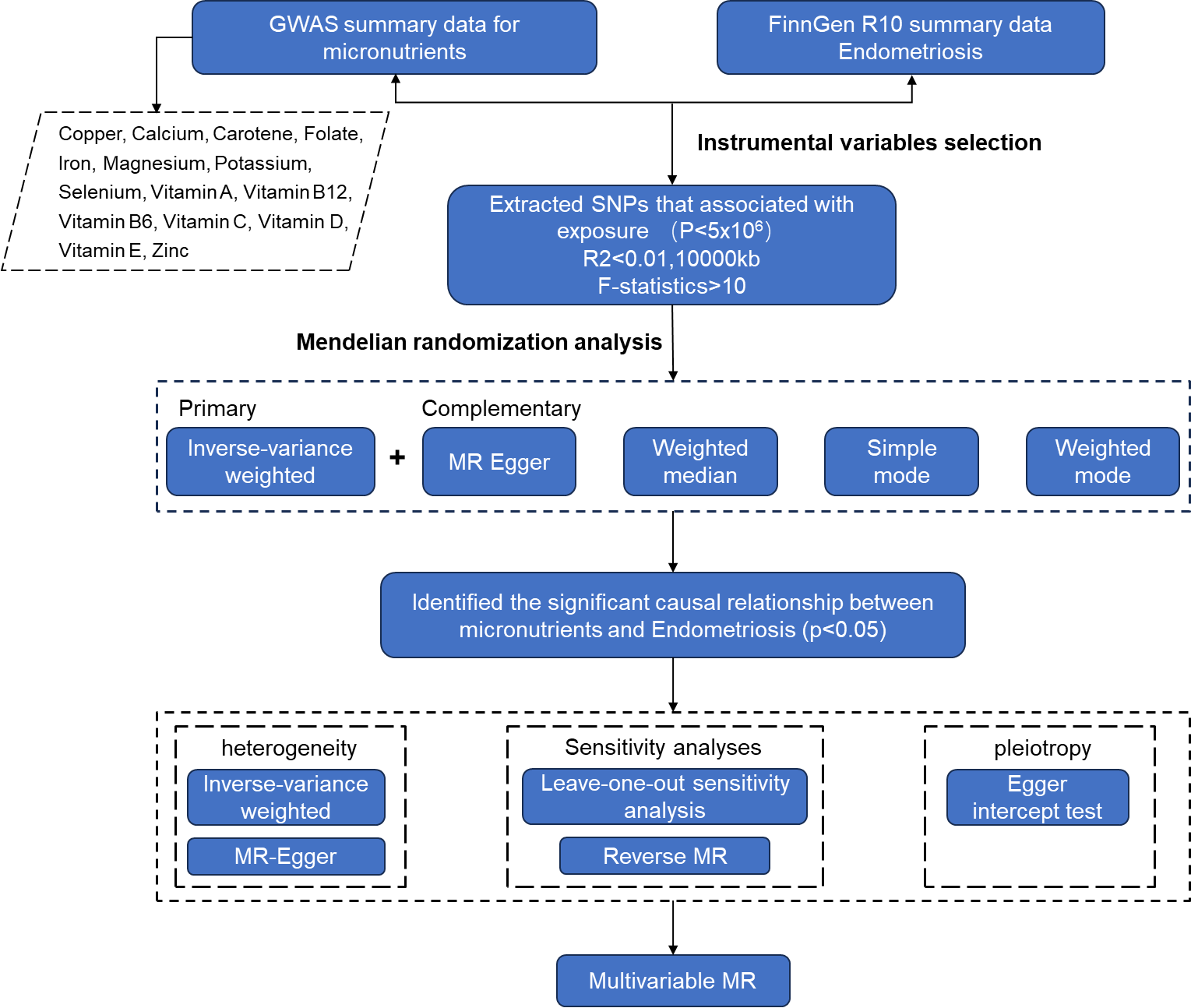


Figure S1 Overall study design and workflow.


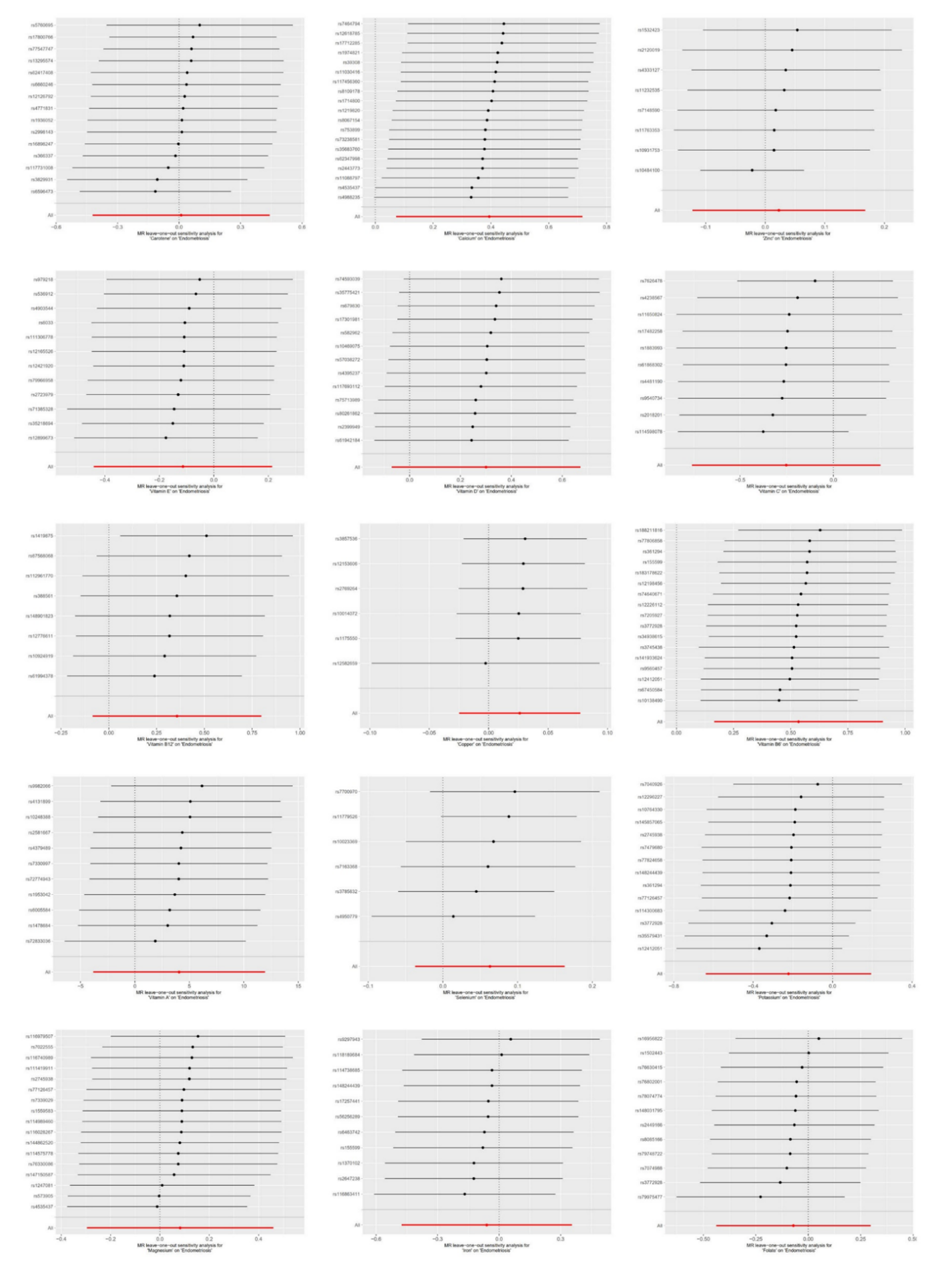
**Figure S2**

Figure S2 Leave-one-out sensitivity analysis results for micronutrients on endometriosis.

**Figure S3**

**
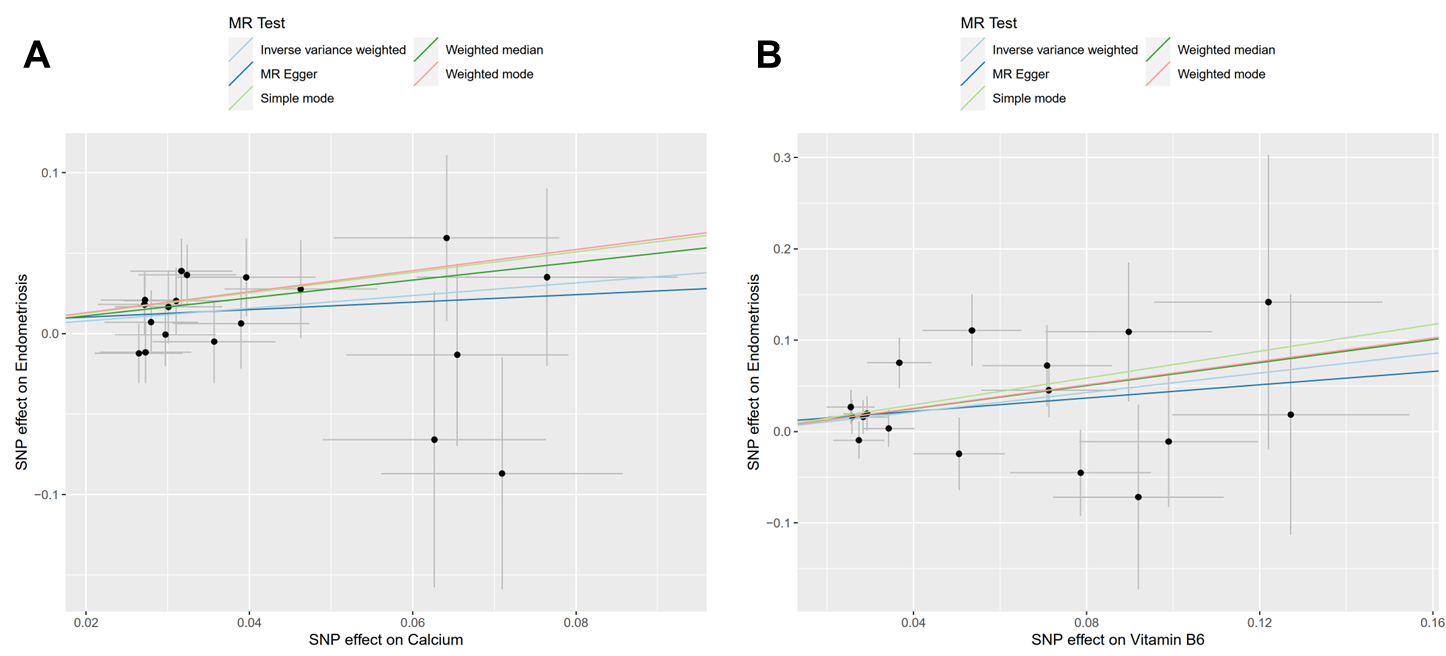
**

Figure S3 Scatter plot of univariate MR analysis results for endometriosis-related micronutrients(A) Calcium, (B) Vitamin B6 on endometriosis.

**Figure S4**


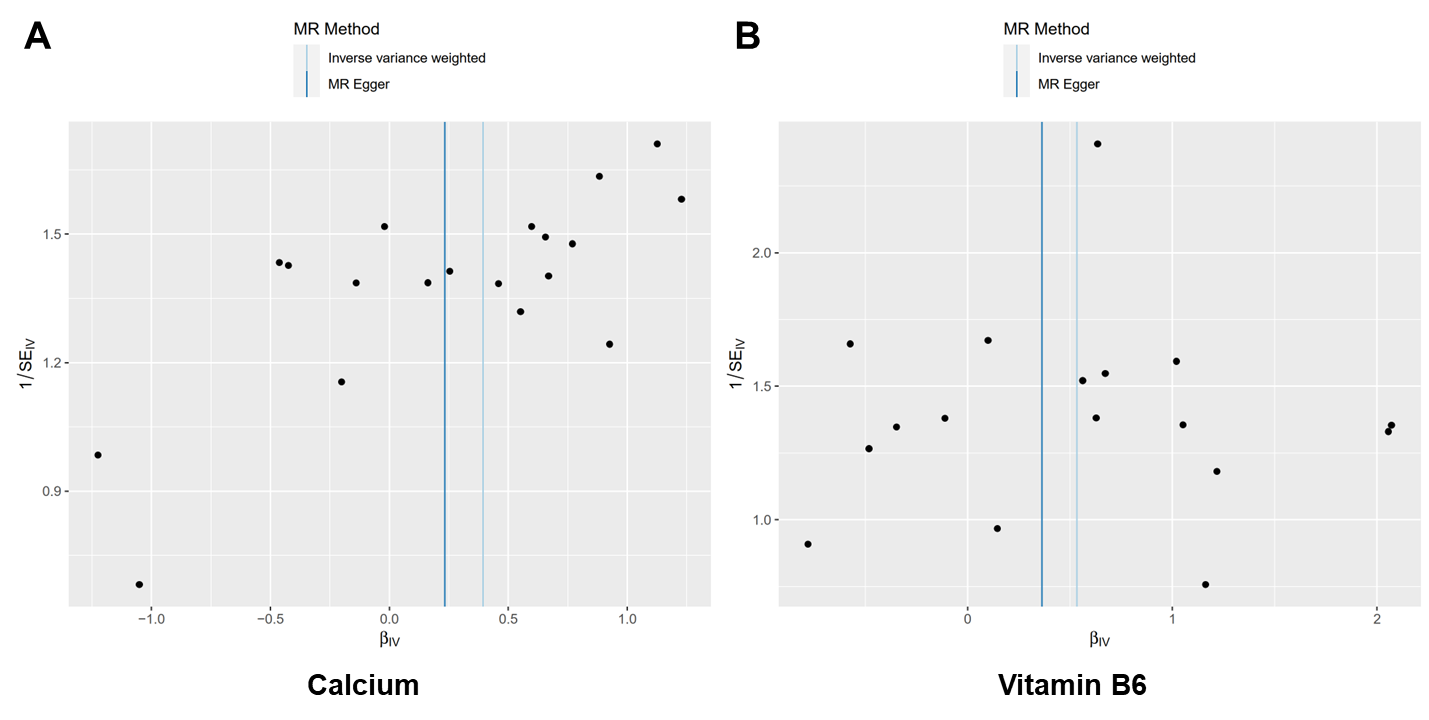


Figure S4 Funnel plot of univariate MR analysis results for endometriosis-related micronutrients(A) Calcium, (B) Vitamin B6 on endometriosis.

**Figure S5**

**
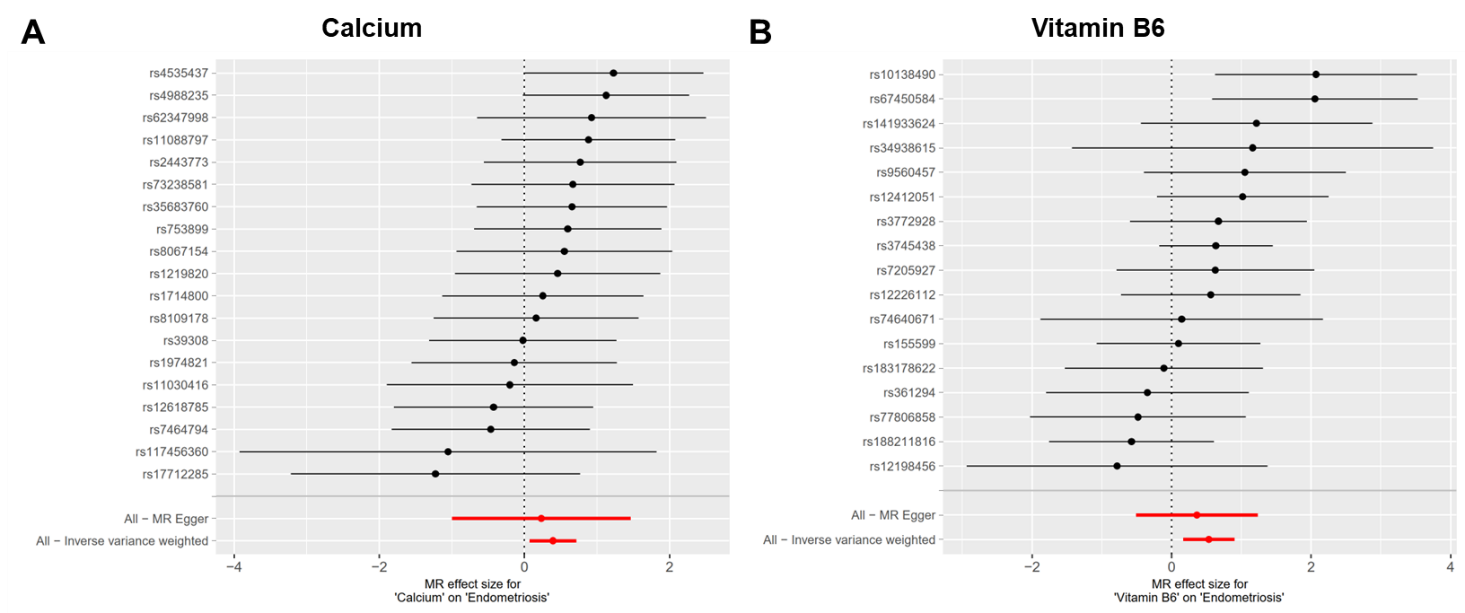
**

Figure S5 Froest plot of individual and combined effect of endometriosis-related micronutrients (A) Calcium, (B) Vitamin B6.

**Figure S6**

**
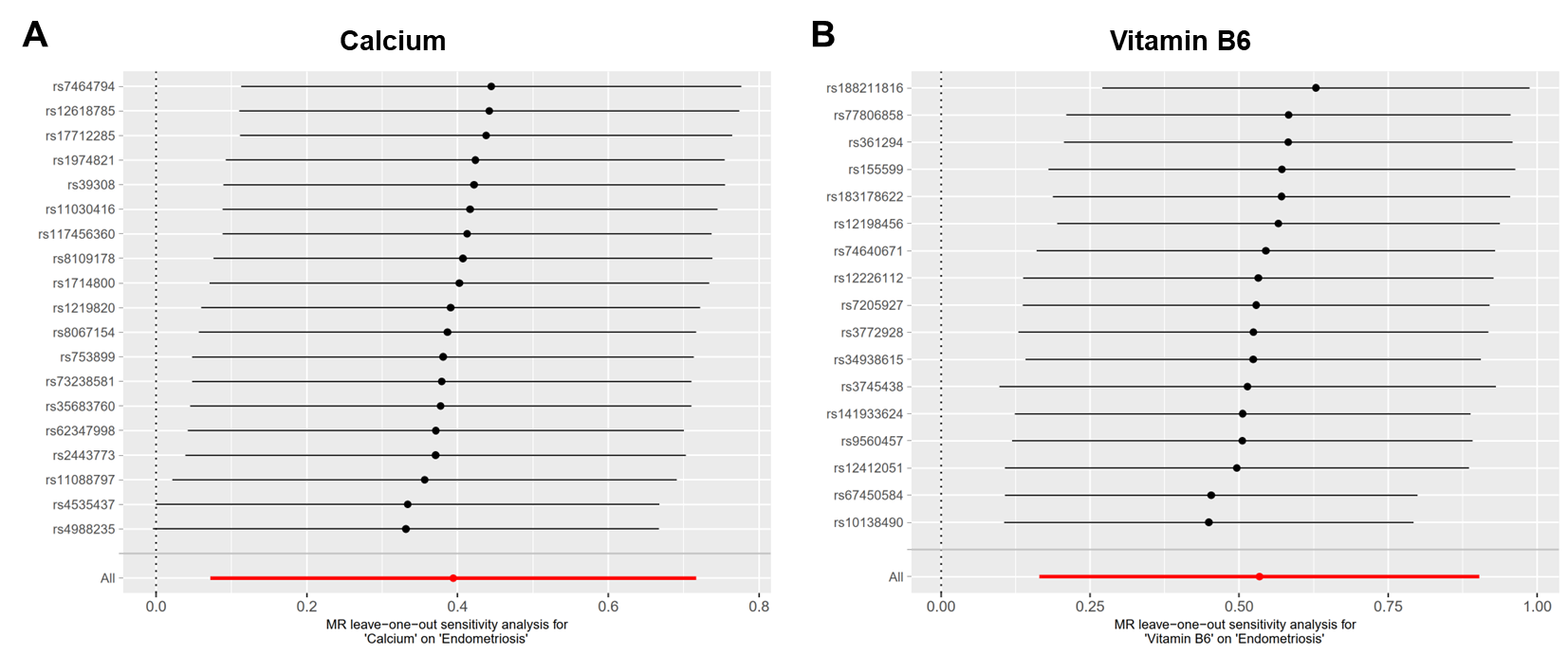
**

Figure S6 Leave-one-out sensitivity analysis results for endometriosis-related micronutrients (A) Calcium, (B) Vitamin B6.

**Supplementary Tables**

**Table S1** Summary information of GWAS database in the two-sample MR study

**Table S2** Information of SNPs selected as instrumental variable of exposure from micronutrients

Table S2 Continued

Table S2 Continued

Table S2 Continued

Table S2 Continued

**Table S3** Univariate MR results for circulating micronutrients and endometriosis

**Table S4** Heterogeneity and pleiotropy analysis for circulating micronutrients and endometriosis in univariate MR

**Table S5** MR analysis of endometriosis-related micronutrients
